# Relationship between Retinal Inner Nuclear Layer Thickness and Severity of Visual Field Loss in Glaucoma

**DOI:** 10.1038/s41598-017-05282-4

**Published:** 2017-07-17

**Authors:** Eun Kyoung Kim, Hae-Young Lopilly Park, Chan Kee Park

**Affiliations:** Department of Ophthalmology and Visual Science, Seoul St. Mary’s Hospital, College of Medicine, The Catholic University of Korea, Seoul, South Korea

## Abstract

Glaucoma is a disease characterized by pathologic changes in inner retinal layers, which are comprised of retinal ganglion cells (RGCs). As retinal ganglion cells (RGCs) cross over other retinal neurons that are connected by synapses, it is meaningful to investigate the outer retinal changes in glaucoma. We evaluated the association between thicknesses of segmented retinal layers in macular region and severity of visual field loss in open-angle glaucoma (OAG). This study involved 103 glaucomatous eyes. Retinal nerve fiber layer (RNFL), ganglion cell layer (GCL), inner plexiform layer (IPL), inner nuclear layer (INL), outer plexiform layer (OPL), and outer nuclear layer (ONL) thicknesses were measured at the macular level using the Spectral-domain optical coherence tomography with segmentation software. The functional losses were measured using 24-2 standard automated perimetry. Macular structure losses were positively correlated with functional loss for RNFL, GCL, and IPL (*R* = 0.550, 0.637, and 0.649, respectively, *P* < 0.001) and negatively correlated with INL (*R* = −0.295, *P* = 0.041). By multivariate regression analysis, INL thickness was significantly associated with visual field mean deviation (dB) and optic disc hemorrhage. These finding carefully suggest reactive responses of neuronal or glial cells located in the INL occur during glaucoma progression.

## Introduction

Glaucoma is a disease characterized by pathologic changes in inner retinal layers, which are comprised of retinal ganglion cells (RGCs). However, the extent to which outer retinal changes occur in glaucoma remains controversial. Several studies have observed structural changes in the outer retinal layer, such as a reduced number of cells in the photoreceptor layer and reported functional impairment in outer retinal neurons using electroretinography (ERG) in primary open-angle glaucoma (POAG) or angle-closure glaucoma (ACG)^1–4^. In contrast, a study of nonhuman primates (NHP) experimental glaucoma has suggested that there is little or no histological evidence of abnormalities distal to the RGC layer^5^.

Despite this controversy, it is meaningful to investigate the outer retinal changes in glaucoma, as RGCs cross over other retinal neurons that are connected by synapses. The dendrites of RGCs form synapses to the bipolar, amacrine, and Müller glial cells located in the inner nuclear layer (INL), and these interneurons connect to the photoreceptors in the outer nuclear (ONL) and outer plexiform layer (OPL). Furthermore, it has frequently been reported that neurons can affect other directly or indirectly synapsed neurons by retrograde or anterograde degeneration^6^. Abbasian *et al*. have shown that unilateral isolated optic nerve hypoplasia, represented by a decrease in the number of axons within the optic nerve, is associated with subclinical outer retinal abnormalities due to trans-synaptic degeneration^7^. Park *et al*. reported that damage to neurons in the visual cortex in the occipital lobe, induced by cerebral infarction, causes retrograde degeneration, which may affect the RGC level^8^.

Advances in imaging equipment and software have allowed more detailed analysis of the retina in the macular region^9, 10^. Spectral-domain optical coherence tomography (SD-OCT) is a noninvasive imaging modality that produces *in vivo* images comparable to histological samples, with good reproducibility^11^. Furthermore, automated segmentation software of SD-OCT facilitates accurate, repeatable, and precise delineation of individual retinal layers^12^.

Although SD-OCT enables quantitative analysis of retinal anatomy, very few studies have evaluated the outer retina in glaucoma. The present study aimed to identify the changes in segmented retinal layers in the macular region and to evaluate structure and function relationships in glaucoma. To this end, we used the segmentation software of the Spectralis SD-OCT (Heidelberg Engineering, Heidelberg, Germany) to measure thicknesses of individual retinal layers at the macular level: retinal nerve fiber layer (RNFL), ganglion cell layer (GCL), inner plexiform layer (IPL), INL, OPL, and ONL.

## Methods

### Study population

This cross-sectional study investigated 103 patients with mild to severe open-angle glaucoma who were enrolled from a clinical database at the glaucoma clinic of Seoul St. Mary’s Hospital, College of Medicine, The Catholic University of Korea, between December 2014 and October 2015. This study was conducted in accordance with the ethical standards stated in the Declaration of Helsinki and with the approval of the Institutional Review Board of Seoul St. Mary’s Hospital. Written informed consent was obtained from consecutive patients who met the eligibility criteria and were willing to participate in the study and publication of identifying images.

As an initial evaluation, all participant underwent a comprehensive ophthalmologic examination, including a review of their medical history, measurement of visual acuity, slit-lamp biomicroscopy, Goldmann applanation tonometry, gonioscopic examinations, central corneal thickness measurement using ultrasound pachymetry (Tomey Corporation, Nagoya, Japan), axial length measurement (IOL Master; Carl Zeiss Meditec, Jena, Germany), dilated stereoscopic examination of the ONH (optic nerve head), stereoscopic optic disc photography, red-free retinal nerve fiber layer (RNFL) photography (VX-10; Kowa Optimed, Tokyo, Japan), Spectralis SD-OCT scans for the measurement of RNFL, GCL, IPL, INL, OPL, and ONL thicknesses in the macular area, and achromatic automated perimetry using the 24-2 Swedish Interactive Threshold Algorithm standard program (Humphrey Visual Field Analyzer; Carl Zeiss Meditec, Inc, Dublin, CA).

The inclusion criteria were: best-corrected visual acuity of 20/30 or better, a spherical equivalent between −6.0 and +4.0 diopters (D) and a cylinder correction within ±3.0 D, presence of a normal anterior chamber and open-angle on slit-lamp and gonioscopic examinations, and reliable visual field (VF) test results, with a false-positive error of less than 15%, a false-negative error of less than 15%, and a fixation loss of less than 33%. Subjects were excluded based on any of the following criteria: a history of retinal disease, including diabetic or hypertensive retinopathy; a history of eye trauma or surgery, with the exception of uncomplicated cataract surgery; a history of ischemic optic nerve disease; a history of systemic or neurological disease that may affect the VF; and any retinal disease, such as diabetic macular edema, epiretinal membrane, and age-related macular degeneration, which can affect macular thickness and induce segmentation error.

Patients were defined as having glaucoma if they had a glaucomatous optic disc appearance (such as focal or diffuse rim thinning, notching, or acquired pitting of the optic nerve) with a visible RNFL defect on red-free photography and glaucomatous VF loss). The stereophotographic assessment was confirmed and consensus was reached by 2 glaucoma specialists (EKK, CKP). Patients with glaucoma were additionally classified into 3 groups based on the severity of their visual field damage; mild glaucoma was defined as visual field mean deviation (MD) higher than −6 decibels (dB), the moderate glaucoma was defined as visual field MD between −6 dB and −12 dB, and severe glaucoma was defined as visual field MD lower than −12 dB^13^.

### Optical Coherence Tomography

**Figure 1:**
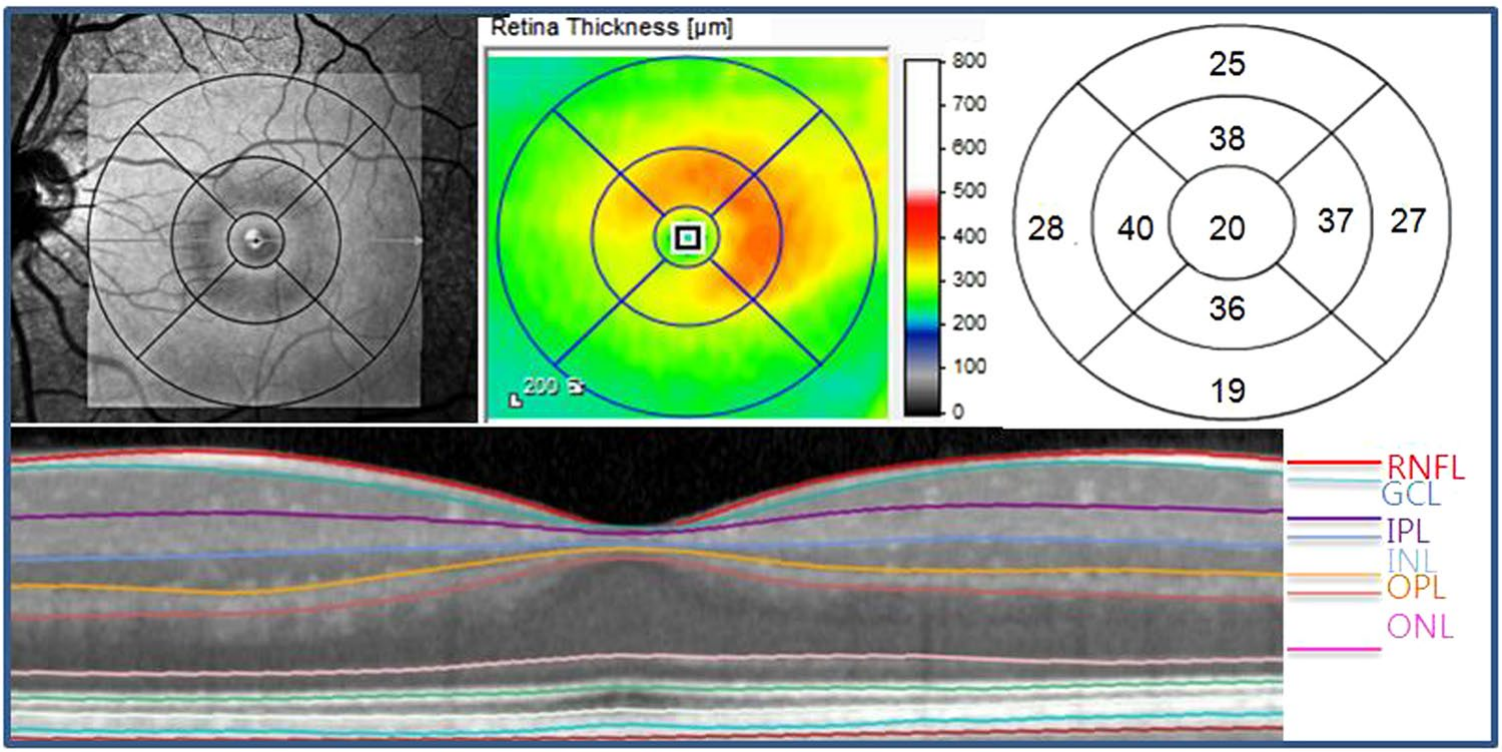
Divisions of the retinal layer using Spectralis SD−OCT. color-coded retinal thickness map showing mean thicknesses for each of nine subfields: scan area of 6 × 6 mm, divided into three concentric circles with 1 mm, 3 mm, and 6 mm diameter, respectively. Here, we used values from the 3 and 6 mm circles of the grid excluding a central area (1 mm radius) that corresponded to the foveola. SD-OCT, spectral-domain optical coherence tomography; RNFL = retinal nerve fiber layer; GCL = ganglion cell layer; IPL = inner plexiform layer; INL = inner nuclear layer; OPL = outer plexiform layer; ONL = outer nuclear layer.

The Spectralis SD-OCT was performed by the same experienced operator on all patients using the fast macular cube scan. The Spectralis SD-OCT uses real-time eye-tracking software (TruTrack; Heidelberg Engineering, Heidelberg, Germany) to center on the patient’s fovea. Images are obtained using 6 macular radial scans with high-resolution, and 20 raster lines, spaced 200 µm apart. During assessment, macular thickness and volume (scan angle: 15 degrees) were determined. The automated segmentation software for the Spectralis OCT uses 6 different retinal boundaries: the inner limiting membrane (ILM), the boundaries between the RNFL and the GCL, between the GCL and the IPL, between the IPL and the INL, between the INL and the OPL, and between the OPL and the ONL. For every OCT scan, each segmented layer line can be manually adjusted and individually shown. Based on the layer segmentations, 6 individual retinal thicknesses were calculated in the macular area: RNFL, GCL, IPL, INL, OPL, and ONL, for each of 9 subfields (Fig. 1). The grid of color-coded retinal thickness map consists of three concentric rings with diameters of 1, 3, and 6 mm. The two outer rings are divided into quadrants by two intersecting lines. At the time of data acquisition, the intermediate and outer rings of diameters 3 and 6 mm, respectively, were considered for the analyses excluding a central area (1 mm radius) that corresponded to the foveola. The intermediate ring is divided into 4 zones, designated as the inner superior, inner nasal, inner inferior, and inner temporal, while the outer ring is divided into the outer superior, outer nasal, outer inferior, and outer temporal zones^14^. The average thickness of each layer was obtained by averaging four inner sectors and four outer sectors. The quality of the scans was assessed before analysis, and well-centered scans and no movement artifacts were included. The eyes were excluded if there was evidence of microcystic changes in any of the retinal layers.

### Visual Field Examination

**Figure 2:**
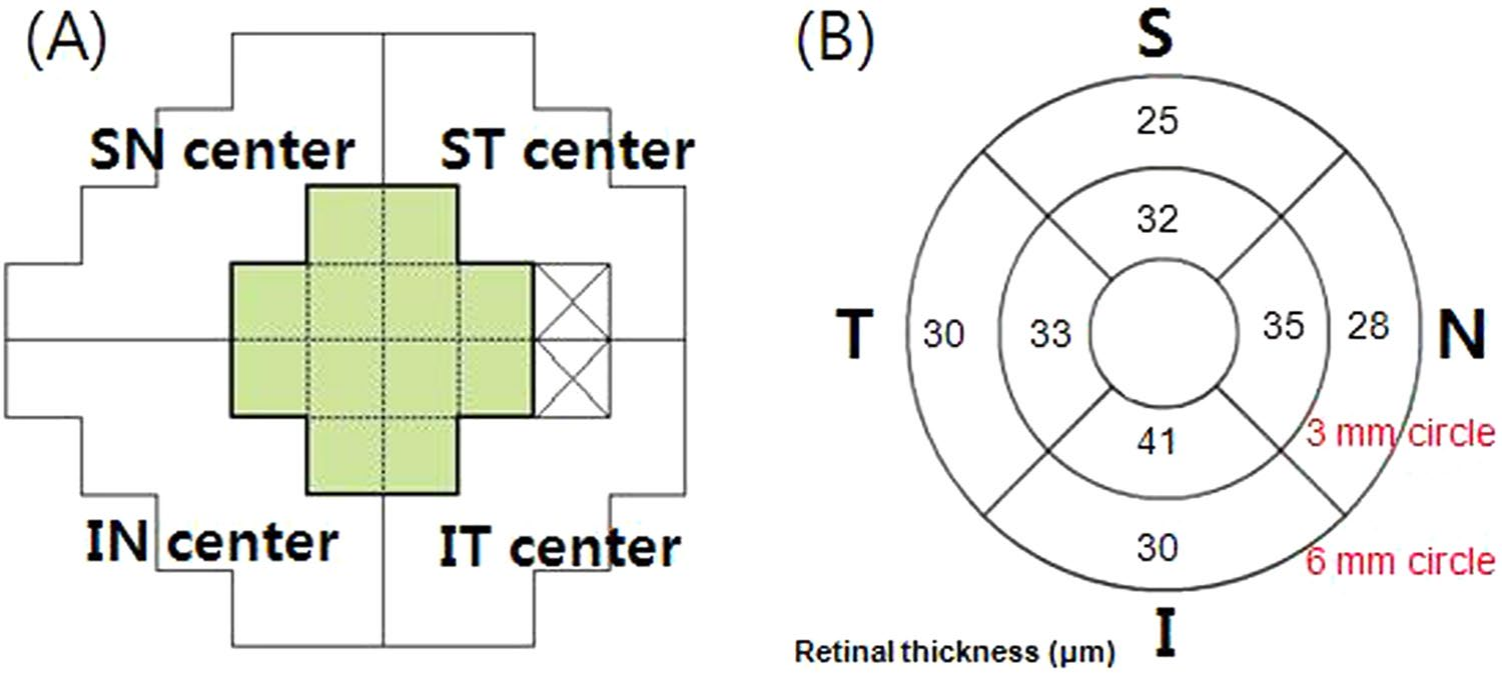
(**A**) The VF of the Humphrey field analyzer Swedish interactive threshold algorithm 24-2 paradigm. The corresponding central cluster MS was defined as the average of 12 central data points. (**B**) segmented retinal layer thickness measurement by Spectralis HD-OCT (scan area of 6 × 6 mm, macular area) of a right eye. S = superior; ST = superotemporal; SN = superonasal; I = inferior; IT = inferotemporal; IN = inferonasal.

All patients underwent VF testing using the Swedish Interactive Threshold Algorithm Standard (SAP) 24-2 strategy on the same day as Spectralis SD-OCT imaging. A glaucomatous VF defect was defined as a cluster of 3 or more points with a probability <5% on the pattern deviation map, including at least 1 point with a probability of <1%; or a result of outside normal limits in the glaucoma hemifield test; or a pattern standard deviation (PSD) with a probability of <5%. Structure–function relationships were analyzed by comparing the corresponding mean sensitivity (MS) values, measured by 24-2 SAP, and the OCT parameters, assessed using SD-OCT. The corresponding area VF MS, which was assumed to correspond topographically within 6.0 mm of the fovea, was defined as the average of 12 central data points (Fig. 2)^15^.

### Statistical Analysis

Statistical analysis was performed using SPSS ver. 16.0 statistical package (SPSS, Chicago, IL). Age, intraocular pressure, central corneal thickness, spherical equivalent, axial length, MD, PSD, and average RNFL, GCL, IPL, INL, OPL, and ONL thicknesses were compared among the mild, moderate, and severe glaucoma groups, using Kruskal–Wallis tests and Bonferroni’s post hoc analysis. Pearson’s correlation analysis was used to evaluate the relationships between thicknesses of RNFL, GCL, IPL, INL, and OPL and VF MS values of the corresponding area. Univariate and multivariate linear regression analyses were performed to find the factors related to changes of segmented retinal layers. Independent variables included were age, systemic diseases, such as diabetes mellitus and hypertension, disc hemorrhage, IOP, CCT, spherical equivalent, axial length, and the MD of VF. Variables with a significance of P < 0.20 in the univariate analysis were included in the multivariate model. A P-value < 0.05 was considered to be statistically significant.

## Results

This study involved 103 eyes with glaucoma. The demographics of subjects with mild to severe glaucoma are summarized in Table 1. The subjects were divided into 3 groups based on MD values and the thickness of the 6 segmented retinal layers were compared (Table 2). There were no marked differences in age, sex, intraocular pressure, central corneal thickness, spherical equivalent, axial length among the 3 groups. Mild glaucomatous eyes had an MD of −3.09 ± 1.72 decibels, moderate glaucomatous eyes −8.80 ± 1.80, and severe glaucomatous eyes −13.69 ± 1.37; these values were significantly different among the 3 groups. The RNFL, GCL, and IPL thicknesses were significantly different among 3 groups. On the other hand, there were no statistically significant changes in the INL, OPL, and ONL thicknesses.

**Table 1:** Demographic and ocular characteristics of participants with glaucoma.

	Glaucoma
Number of subject eyes	103
Mean age, years	53.12 ± 13.97
Gender ratio, male:female	48:55
Intraocular pressure, mmHg	14.35 ± 3.10
Central corneal thickness, μm	536.37 ± 34.47
Spherical equivalent, diopters	−2.27 ± 3.85
Axial length, mm	24.88 ± 1.33
Mean MD of 24-2 VF, dB	−3.84 ± 3.97
Mean PSD of 24-2 VF, dB	4.86 ± 2.28

Data are presented as the mean and standard deviation.

MD = mean deviation; PSD = pattern standard deviation; VF = visual field.

**Table 2:** Average RNFL, GCL, IPL, INL, OPL, and ONL thicknesses in the macular area of glaucoma patients using Spectral-domain optical coherence tomography.

	Mild	Moderate	Severe	*P* value	*P* value		
	Glaucoma (A)	Glaucoma (B)	Glaucoma (C)		Post hoc comparison		
	N = 71	N = 19	N = 13		A-B	A-C	B-C
Average RNFL thickness	26.32 ± 7.38	19.00 ± 2.62	18.80 ± 2.08	0.001	<0.001	<0.001	0.032
Average GCL thickness	35.80 ± 6.93	26.54 ± 5.69	25.30 ± 9.16	<0.001	<0.001	0.001	0.002
Average IPL thickness	30.16 ± 3.68	25.04 ± 2.63	24.60 ± 4.52	<0.001	<0.001	<0.001	0.001
Average INL thickness	36.98 ± 3.60	38.21 ± 2.67	38.60 ± 2.07	0.298	0.313	0.278	0.485
Average OPL thickness	30.30 ± 5.53	29.75 ± 4.35	30.70 ± 10.09	0.612	0.875	0.769	0.908
Average ONL thickness	60.42 ± 8.80	60.58 ± 6.89	60.80 ± 11.29	0.994	0.980	0.984	0.997

RNFL = retinal nerve fiber layer; GCL = ganglion cell layer; IPL = inner plexiform layer; INL = inner nuclear layer; OPL = outer plexiform layer; ONL = outer nuclear layer.

*Comparison among the three groups by Kruskal-Wallis One-way analysis of variance.

**Figure 3:**
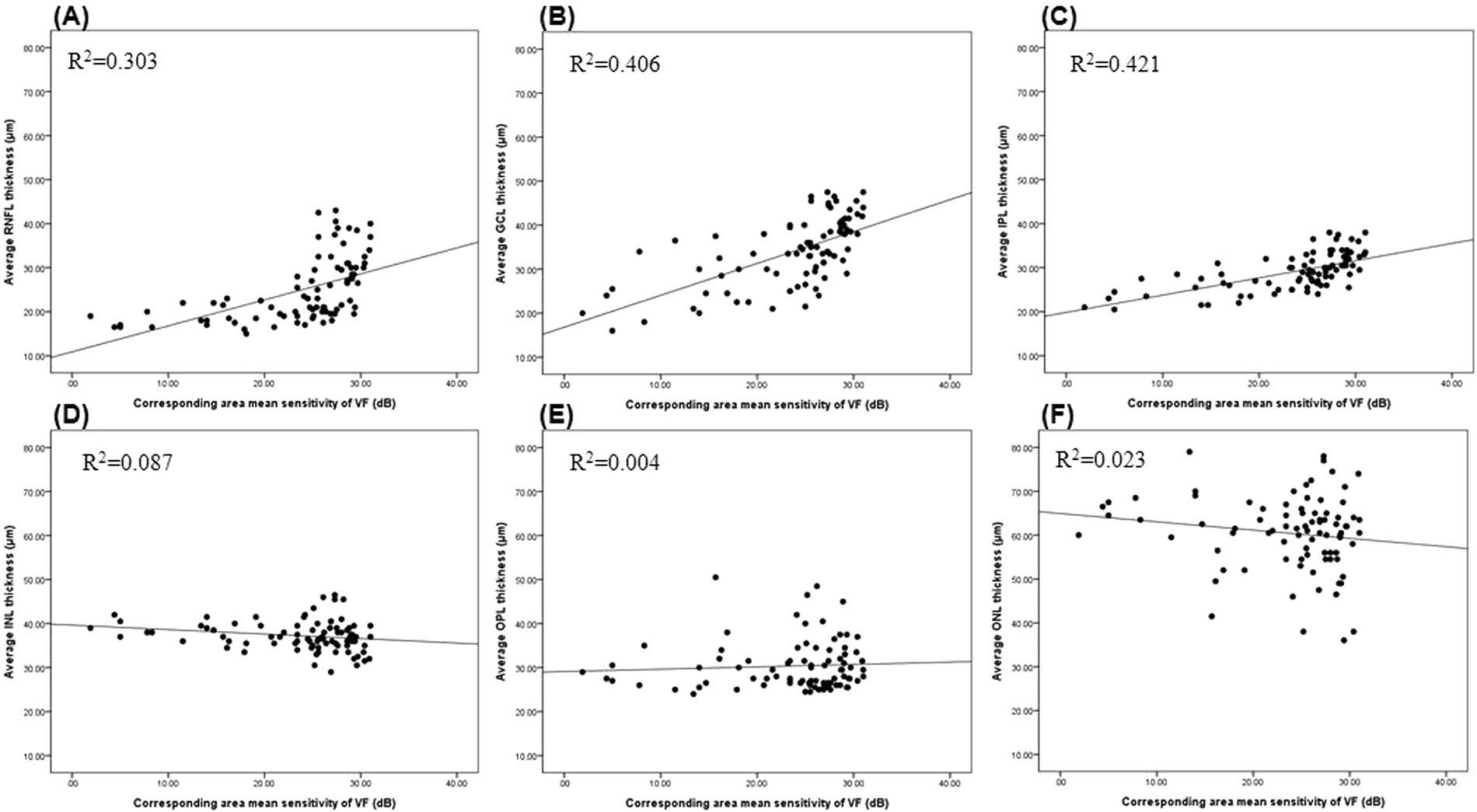
Scatterplots illustrating the linear correlation between standard automated perimetry (SAP) mean sensitivity (MS) value (dB) and spectral domain optical coherence tomography (SD-OCT) segmented retinal thicknesses in the macular area. (**A**) Average RNFL thickness versus corresponding area MS (R^2^ = 0.303, p < 0.001). (**B**) Average GCL thickness versus corresponding area MS (R^2^ = 0.406, p < 0.001). (**C**) Average IPL thickness versus superior corresponding area MS (R^2^ = 0.421, p < 0.001) (**D**) Average INL thickness versus corresponding area MS (R^2^ = 0.087, p = 0.006). (**E**) Average OPL thickness versus corresponding area MS (R^2^ = 0.004, p = 0.553). (**F**) Average ONL thickness versus corresponding area MS (R^2^ = 0.023, p = 0.169). RNFL = retinal nerve fiber layer; GCL = ganglion cell layer; IPL = inner plexiform layer; INL = inner nuclear layer; OPL = outer plexiform layer; ONL = outer nuclear layer.

Table 3 shows the structure and function correlations of segmented retinal layer thicknesses in patients with glaucoma. Macular structure losses were positively correlated with the corresponding MS value of the VF for RNFL, GCL, and IPL thicknesses (R = 0.550, 0.637, and 0.649, respectively, P < 0.001) and negatively correlated with INL thickness (R = −0.295, P = 0.041). These data showed that the relative degree of thickening in the INL is related to the severity of glaucomatous damage as indicated by the loss of MS of the VF. Figure 3 shows the scatter plot and regression analysis of the average thickness of each layer. The RNFL, GCL, and IPL thicknesses (R^2^ = 0.303, 0.406, and 0.421, respectively, P < 0.001) were positively correlated with the MS values of VF and negatively correlated with the INL thickness (R^2^ = 0.087, p = 0.006).

**Table 3:** Pearson correlation coefficient on segmented retinal layer thicknesses in the macular area and corresponding visual field sensitivities*.

	Correlation coefficient	*P* value
Average RNFL thickness	0.550	<0.001
Average GCL thickness	0.637	<0.001
Average IPL thickness	0.649	<0.001
Average INL thickness	−0.295	0.041
Average OPL thickness	0.065	0.553
Average ONL thickness	−0.150	0.169

*Corresponding mean sensitivity value (dB) was measured by 24-2 SAP.

RNFL = retinal nerve fiber layer; GCL = ganglion cell layer; IPL = inner plexiform layer; INL = inner nuclear layer; OPL = outer plexiform layer; ONL = outer nuclear layer.

Table 4 shows correlations between segmented retinal layers. RNFL, GCL, and IPL thicknesses were negatively correlated with INL thickness (R = −0.407, −0.405, and −0.417, respectively, P < 0.001). Logistic regression analysis was performed to determine the factors that are related INL thicknesses (Table 5). Disc hemorrhage and VF mean deviation were identified as significant factors in INL thickening in glaucomatous eyes.

**Table 4:** Pearson correlation coefficient on segmented retinal layer thicknesses in the macular region of glaucoma patients.

Segmented retinal layers	RNFL	GCL	IPL	INL	OPL	ONL
RNFL	1					
GCL	0.769 (<0.001)	1				
IPL	0.728 (<0.001)	0.946 (<0.001)	1			
INL	−0.407 (<0.001)	−0.405 (<0.001)	−0.417 (<0.001)	1		
OPL	−109 (0.321)	−0.006 (0.956)	0.029 (0.789)	0.112 (0.307)	1	
ONL	0.024 (0.825)	−0.116 (0.291)	−0.139 (0.204)	−0.135 (0.218)	−0.906 (< 0.001)	1

RNFL = retinal nerve fiber layer; GCL = ganglion cell layer; IPL = inner plexiform layer; INL = inner nuclear layer; OPL = outer plexiform layer; ONL = outer nuclear layer.

**Table 5:** Association Between INL thicknesses and demographic, ocular variables, and visual field mean deviation: Univariable and multivariable analysis.

Variables	Univariate			Multivariate		
	β	CI	*P* value	β	CI	*P* value
Age	0.083	(−0.001, 0.001)	0.452			
Sex	0.087	(−0.014, 0.033)	0.428			
DM	0.014	(−0.047, 0.053)	0.901			
HBP	0.191	(−0.003, 0.059)	0.080	0.041	(−0.025, 0.037)	0.704
Disc hemorrhage	0.283	(0.010, 0.063)	0.009	0.290	(0.011, 0.064)	0.007
Baseline IOP	0.197	(0.000, 0.005)	0.070	0.234	(0.000, 0.006)	0.025
Mean IOP	0.058	(−0.003, 0.005)	0.598			
CCT	0.068	(0.000, 0.000)	0.548			
Spherial equivalent	−0.040	(−0.004, 0.003)	0.734			
Axial Length	−0.135	(−0.013, 0.004)	0.295			
Visual Field (MD)	−0.290	(−0.007, −0.001)	0.007	−0.311	(−0.007, −0.002)	0.002

INL = inner nuclear layer; DM = diabetes mellitus; HBP = hypertension; IOP = intraocular pressure; CCT = central corneal thickness; MD = mean deviation; β = estimated regression coefficient, CI = confidence interval.

## Discussion

In this study we used SD-OCT scans to measure changes of segmented retinal layer thicknesses in glaucoma patients. A significant contribution of this study is that it focused on outer retina, in which RGCs are not present, in glaucomatous eyes. Surprisingly, in structure and function analysis, we found that macular functional loss is negatively correlated with INL thickness in glaucoma. Very few studies have evaluated outer retina in human glaucoma. Wang *et al*. reported that there were no changes of outer retina, which is a combination of INL and photoreceptor layer in glaucomatous group^10^. Ishikawa *et al*., however, reported that the outer retinal complex was thicker in glaucomatous eyes than in normal eyes^16^. Another study for evaluating structure and function in macular area of NHP experimental glaucoma, macular functional loss was inversely correlated with outer retinal thickness^17^.

In this study, we also observed predictable losses of the macular inner retinal layer thicknesses (RNFL, GCL, IPL), and found that these thickness losses correlated with the MS value of the VF, which itself represented the severity of glaucoma. This result supports previous studies, which have shown that macular inner retinal thicknesses were proportional to loss of peripapillary RNFL thickness and degree of glaucoma^18–23^.

As far as we know, this is the first study to evaluate structure and function of segmented retinal layers including outer retina in human glaucoma. In the present study, the thickness of the INL negatively correlated with the degree of glaucoma (Table 3). Anatomically, the INL consists of bipolar cells and amacrine cells, which are directly synapsed to RGCs, and Müller glial cells, which are involved in retinal environment homeostasis. One possible explanation for this result may be that Müller glial cell hypertrophy is induced by retinal injury associated with glaucoma. Bringmann reported that Müller glial cells respond to retinal damage by changing their morphology, and that these reactive changes can be beneficial to neurons^24^. Goldman also mentioned that glaucomatous or ischemic retinal injury could induce Müller glial cell activation, thus there might be changes on INL thickness^25^. Hesegaya *et al*. referred that macular microcystic edema (MME) was observed at the level of INL in some POAG eyes; however, in our study, we did not observe microcystic changes in the INL^26^.

In this study, we found that RNFL, GCL, and IPL thicknesses were negatively correlated with INL. To explain this correlation, we have analyzed the clinical factors related INL thicknesses (Table 5). Interestingly, VF mean deviation and disc hemorrhage, which known as a prominent characteristic of glaucoma progression were identified as significant factors in INL thickening by multivariate logistic regression. Numerous studies have reported that disc hemorrhage may develop due to vascular dysregulation causing hypoxic retinal injury^27–30^. Also it is well known that various retinal injuries, such as ischemia or glaucomatous damage can induce Müller glial cell activation as a mechanism to repair the damaged retina^31–33^. Thus we carefully suggest that strong correlation between INL thickness and disc hemorrhage or degree of glaucoma could be possibly explained by the reactive response of neuronal or glial cells located in the inner nuclear layer.

While we observed negative correlation between INL thickness and each of 3 inner retinal layers, the INL did not appear to be associated with glaucoma stage. Even though there was not statistically significant, as glaucoma progressed the INL thickness gradually increased. INL thickness showed low variability among the measurements of this layer and the amount of change between mild and severe glaucoma was not that great. For these reasons, we could not find the statistical difference among 3 glaucoma groups regarding INL thickness.

Our study has several limitations. First, artifacts or vitreous detachment can cause an error in automated segmentation, by inducing local thickening of the retina in the macular area. Additionally, a thin RNFL in cases of advanced glaucoma, as well as loss of border clarity, will create difficulties in the use of automated algorithms. To minimize segmentation errors, we verified the segmented layer manually and repeated the segmentation. Second, for structure−function evaluations using retinal thickness in the macular area, the central 10-2 VF test may be more appropriate than 24-2 SAP. To resolve this issue, we selected 12 central VF points using 24-2 SAP, which provided more detailed information on the macular region^34^. Third, in our study population, more than half of the patients were visual field mean deviation higher than −6 dB, the number of eyes defined as severe glaucoma group was quite small compared with mild or moderate glaucoma group. For further study, a greater number of subjects including healthy individuals is needed to obtain more information about structure–function relationships. Finally, this was a cross-sectional study, and could not reveal longitudinal structural and functional data. Therefore, a longitudinal study should be conducted in future, to correlate retinal changes in the macular region with functional status in glaucomatous optic neuropathy.

In summary, we found macular functional losses are positively correlated with RNFL, GCL, and IPL thicknesses specific to RGCs and negatively correlated with INL thickness in glaucoma. INL thicknesses were significantly associated with disc hemorrhage and severity of glaucomatous functional loss.
